# Influence of socioeconomics and social marketing on smoking in Thailand: A National Survey in 2017

**DOI:** 10.18332/tpc/169501

**Published:** 2023-09-01

**Authors:** Pittaya Thammawongsa, Wongsa Laohasiriwong, Nuttapol Yotha, Ampawan Nonthamat, Nakarin Prasit

**Affiliations:** 1 Faculty of Public Health, Khon Kaen University, Khon Kaen, Thailand; 2 Bangkok Hospital KhonKaen, Khon Kaen University, Khon Kaen, Thailand

**Keywords:** smoking behavior, social marketing, alcohol advertisement

## Abstract

**INTRODUCTION:**

Smoking is one of the risk factors for noncommunicable diseases and is harmful to both active and passive smokers. This study aimed to identify the influence of socioeconomic and environmental issues on smoking in Thailand.

**METHODS:**

The study is a secondary dataset analysis of cross-sectional data using data from the 2017 Smoking and Drinking Behaviors Survey of the National Statistical Office of Thailand. The survey collected the data among 88689 participants using a structured questionnaire. The multi-level analysis was used to identify the association between socioeconomics, environmental factors, social marketing, and smoking while controlling for the effects of covariates and presenting the adjusted odds ratio (AOR) and its 95% confidence interval (CI).

**RESULTS:**

Among 88689 respondents, the prevalence of smoking was 18.2% (95% CI: 18.00–18.51). Factors that were associated with smoking were: exposure to secondhand smoke in residential settings (AOR=15.31; 95% CI: 14.47–16.20) and alcohol regular drinking (AOR=4.44; 95% CI: 4.14–4.76). In addition, social marketing factors include: disagreeing or being unsure of the opinions that cigarettes should be categorized as harmful goods (AOR=3.15; 95% CI: 2.94–3.37); not having been exposed to the disadvantages of smoking in social media (AOR=1.51; 95% CI: 1.43–1.61); not having been exposed to the disadvantages of smoking in newspapers, television, radio, advertisements, or other sources (AOR=1.46; 95% CI: 1.37–1.62); having never seen the warning cautions or having seen them but ignored the hazardous effect (AOR=4.81; 95% CI: 4.5–4.9); and having ever seen the warning cautions/ever seen but ignore the hazardous effect (AOR=4.81; 95% CI: 4.54–5.09), and ever seen advertisements or billboards which motivate smoking in various places (AOR=1.33; 95% CI: 1.24–1.42).

**CONCLUSIONS:**

Smoking and secondhand smoke are crucial problems that affect health. In addition, related sectors should help to develop a policy recommendation to reduce the smoking rate through social marketing. Strict and comprehensive policies and laws on non-smoking in work places, public spaces, and homes, will help to reduce secondhand smoke exposure among non-smokers.

## INTRODUCTION

Smoking is one of the risk behaviors causing many diseases that are claiming lives around the world. The World Health Organization estimates that people are dying from smoking-related diseases at a rate of 20000 per day , of which 87.5% die from smoking directly and 12.5% from inhaling secondhand smoke. If the smoking situation remains as is, it is estimated that the death rate will rise to 10 million per year by 2030^1^. Therefore, the World Health Organization introduced the WHO Framework Convention on Tobacco Control (FCTC), an international law of health to solve smoking-related health problems of people and to control the sale of tobacco products, which targets new smokers^2^.

Thailand has joined as a state party and aims to decrease the smoking rate by at least 30% by 2025 (following the goal of decreasing deaths from chronic noncommunicable diseases and completing the durable development goal in the next 15 years)^3^. In Thailand, smoking behavior is second among the risk factors causing diseases. The number of deaths due to smoking-related problems is more than 50000 per year (12% of all causes of death). The diseases caused by smoking are chronic obstructive pulmonary disease (COPD) (23.4%), lung cancer (23.1%), cardiovascular disease (23.0%), and other cancers (14.2%)^4^. With regard to economic loss, which is estimated at 52.2 billion THB (1000 Thai Baht about US$29) , or 0.5% of the gross domestic product (GDP), the economic burden is calculated at 13% of health costs and 73% of the Department of Health budget^5^. The prevalence of smoking among those aged ≥15 years in 2014 and 2015, was 20.7% and 19.9%, respectively. Nevertheless, people are still exposed to secondhand smoke at public places such as markets and pubs which are declared non-smoking areas by law^6^.

Applying the marketing concept in action planning emphasizes influencing people to change knowledge, mindset, and behaviors among individuals, groups, and societies. So, marketing policy should decrease smoking behaviors in Thailand by publishing knowledge inside the commercial departments with health warning pictures on the packaging of cigarettes, making customers aware of the disadvantages of smoking for their health, making people aware of the adverse effects of cigarettes, and creating awareness among the people about health problems^7,8^. This study focused on analyzing and finding the marketing influence on social behaviors while controlling for other factors. The objective of this study was to specify the prevalence of smoking and the influence of marketing on society in Thailand.

## METHODS

### Study design

This study was secondary dataset analysis of the cross-sectional Smoking and Drinking Behavior Survey 2017, organized by the National Statistical Office of Thailand. The survey was used to select participants from representative households from all 77 provinces, representing the entire population. In collecting data from the National Statistical Office’s smoking and drinking behavior survey, using interviews with the head of the household or household members and quality control, the staff gathered data by holding a video conference meeting to clarify the specifics of fieldwork, such as questionnaires, research scopes, etc. The study sample consisted of 129440 participants. In this study, the inclusion and exclusion criteria were set as follows: 1) people aged ≥15 years; 2) representatives of the household; and 3) being a participant in the smoking and drinking survey of the National Statistical Office 2017. The exclusion criteria were as follows: 1) did not have a clear house registration address; and 2) the questionnaire had incomplete information on smoking behavior. Based on the inclusion and exclusion criteria, the total number of participants in this study was 88689.

### Dependent and independent factors

The dependent variable of this study was smoking status (smoker/non-smoker). The independent variable was social marketing, which included opinions on smoking topics, information, the extension of the cigarette trade, advertisements, and a warning on the package as images. Other covariates were gender, age, education level, marital status of parents, income, alcohol consumption, municipality area, and secondhand smoke exposure.

### Data analysis

All the data were analyzed using Stata version 10.0 (Stata Corp., College Station, TX). Descriptive statistics, including frequency and percentage, were used to describe categorical data, whereas mean and standard deviation, or median and range, were calculated for continuous data. A simple logistic regression was used to identify the association between each independent variable and smoking dependence. The independent factors that had a p<0.25 were processed for the multivariable analysis using the multilevel mix-effected logistic regression^9^ to identify the association between all determinants and smoking when controlling for the other covariates. Mixed-effects modeling was used to model fixed effects and random effects of regional and provincial scale. Random effects are useful for modeling intra-cluster correlation since they can reduce cluster-level random effects^10,11^. The magnitude of the effect describing the socioeconomics and social marketing association with smoking in Thailand is presented as an adjusted odds ratio (AOR) with a 95% confidence interval (CI). A value of p<0.05 was considered as a statistically significant level.

## RESULTS

Among the 88689 respondents, 54.2% were female, and 31.3% were aged 45–59 years. Most of them were married (64.9%), and 50.4% lived in a municipality. The highest proportion finished elementary school (41.9%). About 70% were employed, with a median (range) monthly income of 16912 THB (0–99999). Referring to the risk behaviors, 15.9% drank alcohol in residential areas, and 40% were exposed to secondhand smoke in residential areas.

In all, 87.5% had ever seen the warnings on the cigarette packages, but 12.5% (12.33–12.76) had never seen the warnings on cigarette packages, and 12.4% (12.23–12.67) disagreed that cigarettes should be categorized as harmful goods. In terms information regarding the adverse effects of smoking, 14.7% (14.51–14.98) obtained it from social media, 25.3% (25.02–25.60) from newspapers, television, radio, and other, and 50.5% (50.18–50.84) acquired the information from conversations.

The prevalence of smoking among those aged ≥15 years in Thailand was: occasional smoker 16.1% (95% CI: 15.94–16.43), former smoker 9.2% (95% CI: 9.03–9.42), current smoker (smoking daily) 2.07% (95% CI: 1.97–2.16), and never smoker 72.5% (95% CI: 72.22–72.81). The prevalence of smoking was higher among males (37.79%) compared to females (1.80%) (Table 1).

**Table 1:** Prevalence of smoking in Thailand (N=88689)

*Smoking status*	*Males n*	*% (95% CI)*	*Females n*	*% (95% CI)*	*Total n*	*% (95% CI)*
Never smoker	17604	43.42 (42.94–43.90)	46714	97.03 (96.87–97.17)	64318	72.52 (72.22–72.81)
Former smoker	7617	18.79 (18.41–19.17)	567	1.18 (1.08–1.28)	8184	9.23 (9.03–9.42)
Occasional smoker	13649	33.67 (33.20–34.13)	705	1.46 (1.36–1.57)	14354	16.18 (15.94–16.43)
Current smoker	1673	4.13 (3.93–4.32)	160	0.33 (0.28–0.39)	1833	2.07 (1.97–2.16)

The multi-level logistic regression analysis indicated that other significant associated factors with smoking were male gender (AOR=32.47; 95% CI: 29.95–35.20), Secondhand smoke exposure (AOR=15.31; 95% CI: 14.47–16.20), drinking alcohol occasionally (AOR=2.72; 95% CI: 2.54–2.91), and drinking alcohol regularly (AOR=4.44; 95% CI: 4.14–4.76), aged 20–59 years (AOR=3.81; 95% CI: 3.37–4.31), aged ≥60 years (AOR=2.70; 95% CI: 2.37–3.09), uneducated (AOR=1.83; 95% CI: 1.67–2.00), pre-elementary school to vocational high school (AOR=2.47; 95% CI: 2.12–2.87), and social marketing factors: awareness and attitude towards smoking that it is harmless (AOR=3.50; 95% CI: 3.21–3.81), not aware of the danger warnings on cigarette packs (AOR=4.47; 95% CI: 4.18–4.79), have not received information about the dangers of smoking on social media (AOR=1.50; 95% CI: 1.38–1.63), and have not received information about the dangers of smoking on newspapers/television/radio/propaganda and other (AOR=1.48; 95% CI: 1.39–1.59). However, tobacco advertising on consumer motivation to smoke in various places were also found to be associated with smoking (AOR=1.15; 95% CI: 1.06–1.25) (Table 2).

**Table 2:** Multivariable analysis of factors associated with smoking in Thailand, using the multilevel model (N=88689)

*Factors*	*Number*	*Smoking %*	*OR*	*AOR*	*95% CI*	*p*
**Social marketing**						
**Cigarette should be categorized as harmful goods**						<0.001
Agree (Ref.)	81700	15.66	1	1	1	
Disagree/unsure	6989	48.52	5.07	3.50	3.21–3.81	
**Warnings on cigarette packs**						<0.001
Ever seen and realized the dangers (Ref.)	77564	13.53	1	1	1	
Never seen/ever seen but not realized the dangers	11125	51.20	2.59	4.47	4.18–4.79	
**Source of information of smoking dangers**						
**Social media**						<0.001
Yes (Ref.)	13076	12.82	1	1	1	
No	75613	19.29	1.61	1.50	1.38–1.63	
**Newspaper/television/radio/propaganda/other**						<0.001
Yes (Ref.)	22450	14.42	1	1	1	
No	66239	19.55	1.44	1.48	1.39–1.59	
**Advertisements or billboards which motivated people to smoke in various places**						<0.001
No (Ref.)	79414	17.58	1	1	1	
Yes	9275	23.97	1.48	1.15	1.06–1.25	
**Sociodemographics**						
**Gender**						<0.001
Female (Ref.)	48146	1.80	1	1	1	
Male	40543	37.79	33.21	32.47	29.95–35.20	
**Age (year)**						<0.001
15–19 (Ref.)	6006	8.96	1	1	1	
20–59	59585	20.84	2.67	3.81	3.37–4.31	
≥60	23098	14.00	1.65	2.70	2.37–3.09	
**Education level**						<0.001
Certificate/diploma to graduate/postgraduate (Ref.)	13313	9.43	1	1	1	
No education	4358	18.08	2.11	1.83	1.67–2.00	
Pre-elementary school to high vocational school	71018	19.91	2.39	2.47	2.12–2.87	
**Monthly income (THB)**						<0.001
>20000 (Ref.)	14462	11.36	1	1	1	
≤20000	74227	19.59	1.90	1.32	1.21–1.43	
**Secondhand smoke exposure at home**						<0.001
No (Ref.)	58547	66.01	1	1	1	
Yes	30142	33.99	10.68	15.31	14.47–16.20	
**Alcohol consumption**						<0.001
Never/formerly (Ref.)	65670	9.37	1	1	1	
Occasionally	13033	32.64	4.69	2.72	2.54–2.91	
Regularly	9986	57.90	13.31	4.44	4.14–4.76	

THB: 1000 Thai Baht about US$29.

## DISCUSSION

Almost one in five (18.2%) of the Thai population was found to be smoking, which shows the declining prevalence trend compared to that of the previous year. In 2013 and 2014, the percentage of smokers was 20.7% and 19.90%, respectively^5^.When compared with the neighboring countries of Thailand, those with lower prevalence than Thailand were Cambodia (16.9%) and Singapore (12%), while other countries such as Laos (27.9%), Myanmar (26.1%), Malaysia (22.8%), and Vietnam (22.5%) had higher prevalence^12^. The declining trend of smoking in Thailand was due to the performance of WHO MPOWER measures for tobacco control. Including monitoring and evaluating the tobacco-free policy, the quit smoking project, health warnings, mass media, a ban on advertising, and taxing. Also observed was that the prevalence of smoking was as high as 37.7%. It is crucial to have a national tobacco control policy in order to achieve the WHO goal of decreasing smoking by 30% within 2025^13^.

From this study, social marketing factors and socioeconomics were associated with smoking behavior in Thailand. Previous research from other countries^14,15^ report that attitude is a very important factor, because it determines the decision on smoking behavior. In addition, attitude is an expression of a person’s behavior caused by perceptions from knowledge, experience, motivation, and the environment that affect the perception of danger from smoking behavior. One of the important factors that will help to change an attitude on smoking are picture warnings on tobacco packages. The warning information should be presented in such a way that it should discourage the beginners and motivate to quit the current smokers^16-18^, and provide knowledge to change a person’s behavior. If a person has the correct knowledge and understanding, it allows the person to perform or express behaviors correctly and appropriately. Environment is also an important factor that can promote and stimulate a person to smoke in different places, which increases the smoking rate^19,20^. Moreover, it is observed that advertisements promote smoking initiation among youth^21^.

When considering sociodemographic characteristics, it was observed that the smoking rate was much higher among males compared to females, being 33 times higher in males than females. This might be due to the fact that women are not allowed to consume tobacco. As well as a belief that smoking is socially acceptable among men^22^. Even though youth are highly prone to practicing smoking behavior, some countries have laws prohibiting the consumption of tobacco-related products, consistent with the findings of a study in north-eastern Thailand^23^. However, tobacco is one of the addictive substances working people are most likely to be exposed to. This can cause addiction to smoking. Such addiction makes it very difficult to quit smoking among working age groups^24,25^. A study’s results revealed that higher education level is associated with a lower smoking rate. Pre-elementary school to high vocational school students had a two-fold higher smoking rate compared to those with higher education level. People with pre-elementary and high vocational school education were mostly working as laborers. The high rate of smoking in this group might be due to a lack of knowledge and awareness of smoking and its adverse effects. Higher education level was associated with an intention to quit smoking^26-28^. Another supporting factor of smoking is alcohol consumption behavior; a study showed that 90.54% of drinkers smoke. The number of smokers is proportional to the frequency of drinking alcohol. It might be the result of alcohol and nicotine being related substances. Once somebody starts to take one of the substances, it might be stimulating to receive another substance. Therefore, consuming alcoholic beverages may increase the interest in smoking^29,30^. Another smoking problem is secondhand smoke exposure, which is associated with demographic characteristics similar to those of active smoking. Previous studies reported that people of low socioeconomic status are exposed to secondhand smoke^31,32^. They found that as high as 33.9% of households were exposed to secondhand smoke, of which 24.9% were exposed every day. They also found that exposure to secondhand smoke in households was able to initiate smoking by 15 times, the same as previous studies that indicated that smoking and secondhand smoke were related^33,34^. Smoking and secondhand smoke exposure are crucial problems that affect health. Strict and comprehensive policies and laws on non-smoking areas in work places, public areas, and homes, will help to reduce secondhand smoke exposure among non-smokers^35,36^.

### Limitations

Secondary data were obtained from the National Statistical Office. Therefore, there are limitations in terms of variables and data integrity. As a result, the analysis in this study was not able to use some of the variables to find the relationship between them. As the study was cross-sectional we are unable to attribute causal associations. Moreover, as the data were self-reported they are potentially subject to response bias.

## CONCLUSIONS

Thailand has tobacco control, such as monitoring and evaluating the tobacco-free policy, the quit smoking project, health warnings, mass media, a ban on advertising, and taxing. Attitude, knowledge, experience, motivation, and environment are important factors that can help change an attitude towards smoking. Picture warnings on tobacco packages are an important factor that can discourage beginners and motivate them to quit. Environment is also an important factor that can promote and stimulate a person to smoke in different places.

Finally, it was observed that the smoking rate was much higher among males compared to females. The law of the country prevents working age groups from consuming tobacco-related products, but youth are still prone to smoking. Lower education level is associated with a higher smoking rate, likely due to lack of knowledge and awareness of the adverse effects of tobacco smoking. Smoking and secondhand smoke exposure are important health issues, with higher education level being associated with an intention to quit smoking. Alcohol consumption is linked to higher smoking rates, and secondhand smoke exposure can be especially dangerous for those of low socioeconomic status. To reduce secondhand smoke exposure, strict policies and laws should be implemented in work places, public areas, and homes.

## Data Availability

The data supporting this research cannot be made available for privacy or other reasons.
